# Supplementary material for: Massively Parallel Profiling of HIV-1 Resistance to the Fusion Inhibitor Enfuvirtide
Source: Viruses. 2019 May 15;11(5):439. doi: 10.3390/v11050439 (PMC6563210; doi:10.3390/v11050439)
Supplement: Supplementary file 1 [file viruses-11-00439-s001.zip › 5.viruses-499401-suppl_/figure S1-S4.pdf]

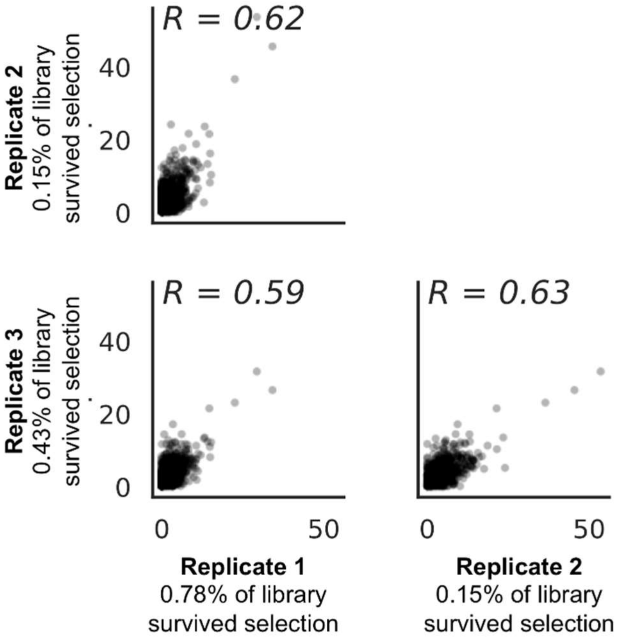

Figure S1. The correlations between the positive site differential selection for biological triplicates. The fraction of the virus that survived enfuvirtide selection, as calculated from a qPCR standard curve, is labeled for each replicate.

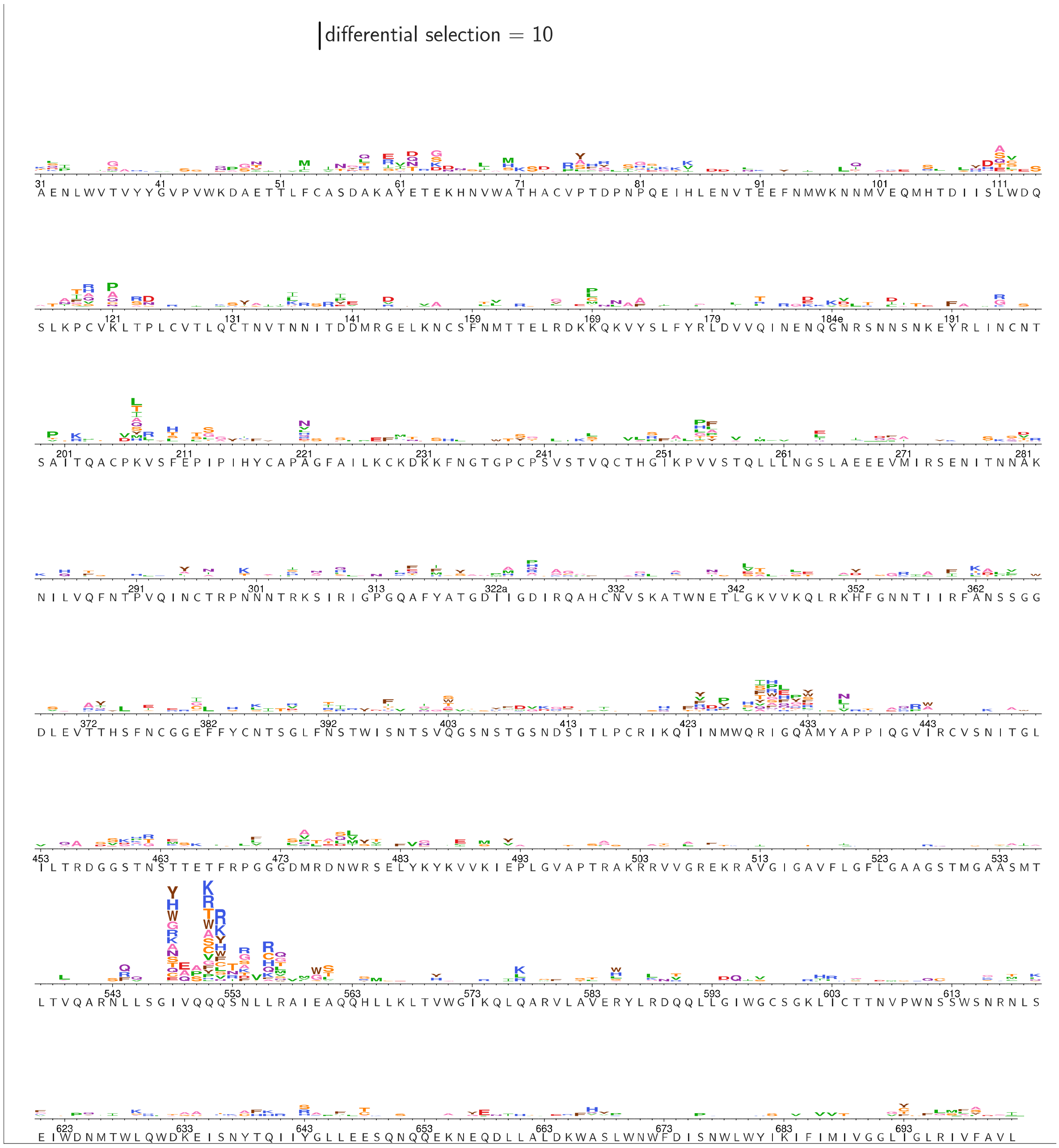

Figure S2. The complete, mutation-level resistance profile across Env. The height of each amino acid is proportional to its differential selection.

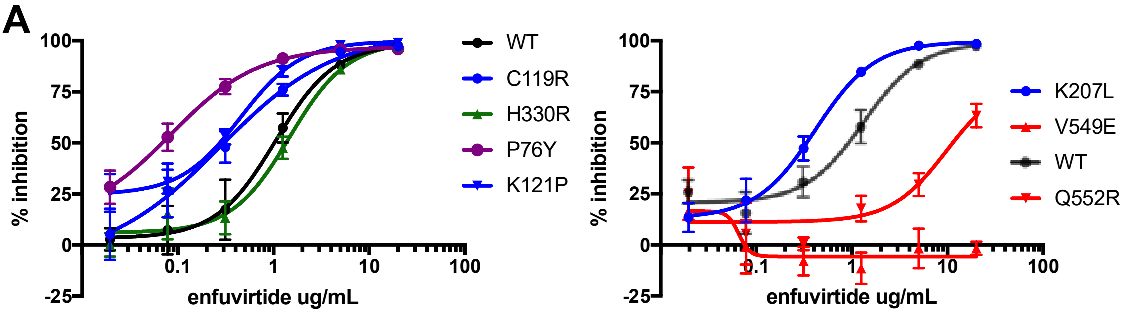

B

plate	Mutant	DMS resistance profiling: differential selection	TZM-bl inhibition assay (10 μg/mL DEAE-dextran): IC50 (μg/mL) mean	IC50 (μg/mL) standard error	Fold change in IC50 mean	Fold change in IC50 standard error	Maximum % inhibition mean	Maximum % inhibition standard error
1	C119R	1.71	0.3035	0.07897	0.29	0.04	107.8	7.65
1	H330R	-1.52	1.3316	0.13507	1.36	0.32	99.7	3.09
1	P76Y	2.53	0.0697	0.00764	0.07	0.00	97.0	0.63
1	K121P	3.37	0.2739	0.01859	0.27	0.02	100.0	0.34
1	WT	-	1.0155	0.14005	1.00	-	98.7	0.04
2	K207L	2.94	0.3377	0.02876	0.36	0.08	99.5	0.15
2	V549E	2.89	>20	-	>25	-	24.5	6.86
2	WT	-	0.9677	0.14323	1.00	-	98.2	0.88
2	Q552R	5.93	10.8560	1.45797	11.24	0.16	76.4	9.22

Figure S3. TZM-bl inhibition assay performed with 10 μg/mL DEAE-dextran instead of the 100 μg/mL concentration used in Figure 3 and resistance profiling experiments. **A**. and **B.** As in Figure 3.

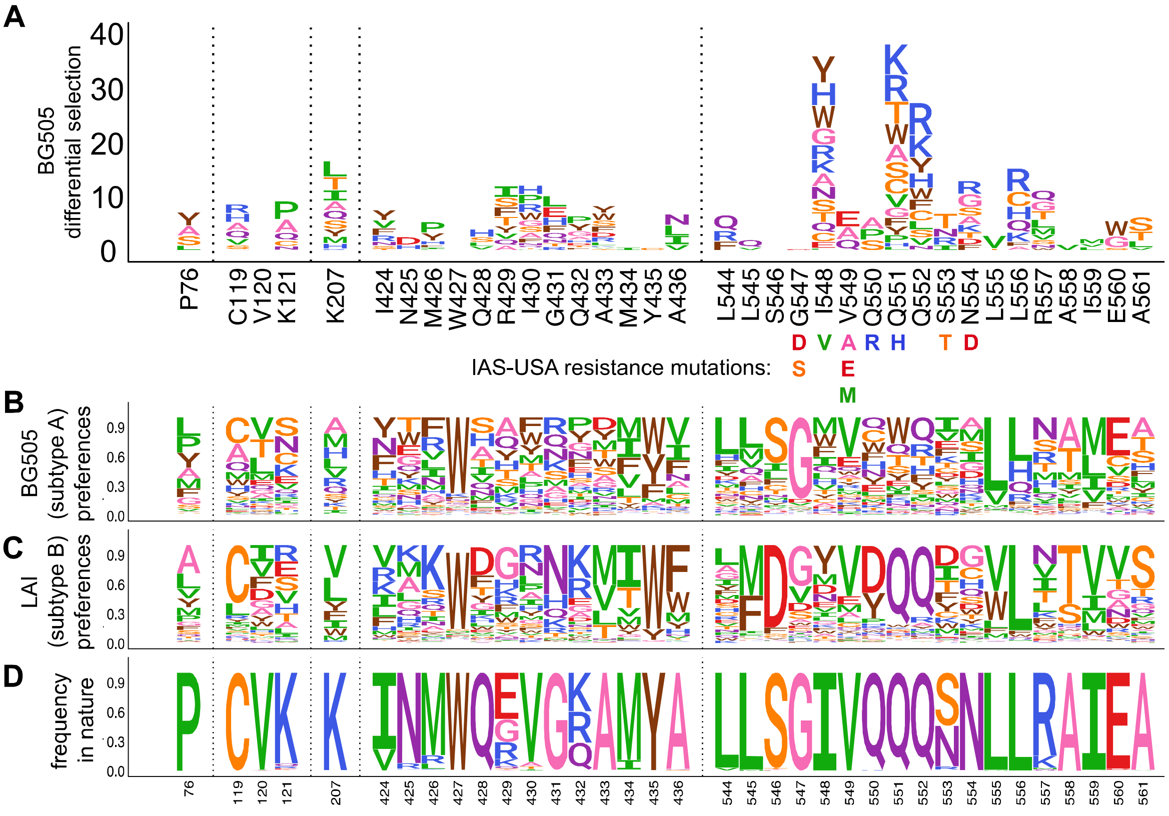

Figure S4. Comparing BG505 enfuvirtide resistance to BG505 (subtype A) mutational tolerance, LAI (subtype B) mutational tolerance, and natural sequence variation **A**. The enfuvirtide resistance profile generated using BG505 mutant virus libraries, as presented in Figure 1B. **B.** The amino-acid preferences for BG505 Env, measured using the same BG505 mutant viral libraries used in the resistance profiling, but exerting selection only for viral replication in SupT1.CCR5 cells. As measured in [34]. Briefly, the height of each amino acid corresponds to how well tolerated that amino acid is for viral replication in cell culture. **C**. The amino-acid preferences for the subtype B LAI Env, under selection only for viral replication in cell culture. As measured in [35]. **D**. The amino-acid frequencies among naturally circulating viral variants, calculated from the group M filtered LANL Web Alignment [49].

49. Foley, B.; Leitner, T.; Apetrei, C.; Hahn, B.; Mizrachi, I.; Mullins, J.; Rambaut, A.; Wolinsky, S.; Korber, B. HIV Sequence Compendium 2017. *Theor. Biol. Biophys. Group:* Los Alamos Natl. Lab., NM, USA **2017**.
