# Supplementary material for: Massively Parallel Profiling of HIV-1 Resistance to the Fusion Inhibitor Enfuvirtide
Source: Viruses. 2019 May 15;11(5):439. doi: 10.3390/v11050439 (PMC6563210; doi:10.3390/v11050439)
Supplement: Supplementary file 1 [file viruses-11-00439-s001.zip › 5.viruses-499401-suppl_/SupplementalFiguresFiles/SupplementalFiles1-2/FileS1.zip!/SupplementaryFile1/analysis_notebook.html]

analysis\_notebook


# Massively parallel profiling of HIV-1 resistance to the fusion inhibitor enfuvirtide¶

### Adam S. Dingens, Julie Overbaugh, and Jesse D. Bloom¶

Experiments analyzed here were performed by Adam Dingens in the Bloom lab and Overbaugh lab in the late 2017 - 2018.

Here, we use slightly modified version of mutational antigenic profiling, which we can refer to as resistance profiling, to comprehensively map resistance to `enfuvirtide`, also known as `T-20` and tradename `Fuzeon`. We use BG505.T332N mutant Env libraries, first described and characterized in Haddox, Dingens et al 2018. The triplicate mutant libraries used here correspond to the three BG505 replicates in this paper.

We use dms\_tools2 to analyze the data . This notebook processes the Illumina deep sequencing data, and then analyzes the enfuvirtide selection.

Dingens et al 2017 describes mutational antigenic profiling in detail. The barcoded subamplicon Illumina sequencing approach is described here, and the computation of `differential selection` is described here.

## Import `Python` modules, define widely used functions¶

Set `use_existing` to `yes` below if you want to use any existing output, and `no` if you want to re-generate all output.

In [1]:

```
import os
import glob
import pandas as pd
from IPython.display import display, HTML
import dms_tools2
import dms_tools2.plot
import dms_tools2.sra
import dms_tools2.utils
import dms_tools2.diffsel
from dms_tools2.ipython_utils import showPDF
import rpy2
import rpy2.robjects 
import dms_tools2.rplot

import numpy as np
import pylab as plt
from colour import Color
print("Using dms_tools2 version {0}".format(dms_tools2.__version__))
# results will go in this directory
resultsdir = './results/' 
if not os.path.isdir(resultsdir):
    os.mkdir(resultsdir)
    
# CPUs to use, -1 means all available
ncpus = 14

# do we use existing results or generate everything new?
use_existing = 'yes'
use_existingalignment = 'yes'
```

```
Using dms_tools2 version 2.3.0
```

## Download the sequencing data from the Sequence Read Archive¶

Here we download sequencing data for each sample from the Sequence Read Archive. Sample names specificy if they are mutant (mut) or wildtype (wt), as well as virus (virus) or DNA (DNA). The *#b* is which replicate. There is metadata available from the SRA, and the code below also links samples names with accession numbers.

They Illumina reads were submitted to the NCBI SRA with SRA accession numbers `SRR8097918 - SRR8097920` on Oct 23, 2018. The BioProject ID is PRJNA498100, with submission number SUB4674031.

This analysis also uses wt virus, mock mutant virus, and wt DNA sequencing that was previously uploaded as part of another paper as SRP157948. Of note, the replicates were performed in experimental parallel at the same time as these previous experiments such that the controls were always processed in parallel.

We download these files using the dms\_tools2.sra.fastqFromSRA function from the dms\_tools2 Python API. Note that the call to this function below uses two external programs that are not part of dms\_tools2, and which you therefore must install externally on the computer that you are using:

1. The fastq-dump program from the SRA Toolkit. If you do not already have this toolkit installed, you will need to install a relatively recent version.
2. The Aspera Connect program for rapid downloads. You need both the executable ascp and an Aspera Key file. Installing Aspera Connect and a key can be somewhat complex, so if you do not want to do this then just set aspera=None in the command below and fastq-dump will do the downloads (albeit more slowly).

In [2]:

```
samples = pd.DataFrame.from_records(
        [('mut-virus-rep1b','SRR7693968'),
         ('mut-virus-rep2b','SRR7694018'),
         ('mut-virus-rep3b','SRR7694003'),
         ('wt-virus-rep1b','SRR7693980'),
         ('wt-virus-rep3b','SRR7693984'),
         ('wt-virus-rep2b','SRR7693970'),
         ('mut-DNA-rep2','SRR7694002'),
         ('mut-DNA-rep1','SRR7693986'),
         ('mut-DNA-rep3','SRR7694021'),
         ('wt-DNA-rep2','SRR7693998'),
         ('wt-DNA-rep1','SRR7693989'),
         ('wt-DNA-rep3','SRR7694017'),
         ('mut-virus-rep1b-T20-8ug','SRR8097920'),
         ('mut-virus-rep2b-T20-8ug','SRR8097919'),
         ('mut-virus-rep3b-T20-8ug','SRR8097918')],
        columns=['name', 'run']
        )


fastqdir = './results/FASTQ_files/'
if not os.path.isdir(fastqdir):
    os.mkdir(fastqdir)
print("Downloading FASTQ files from the SRA...")
dms_tools2.sra.fastqFromSRA(
        samples=samples,
        fastq_dump='fastq-dump', # valid path to this program on the Hutch server
        fastqdir=fastqdir,
        aspera=(
            '/app/aspera-connect/3.5.1/bin/ascp', # valid path to ascp on Hutch server
            '/app/aspera-connect/3.5.1/etc/asperaweb_id_dsa.openssh' # Aspera key on Hutch server
            ),
        )
print("Here are the names of the downloaded files now found in {0}".format(fastqdir))
display(HTML(samples.to_html(index=False)))
```

```
Downloading FASTQ files from the SRA...
Here are the names of the downloaded files now found in ./results/FASTQ_files/
```

| name | run | R1 | R2 |
| --- | --- | --- | --- |
| mut-virus-rep1b | SRR7693968 | mut-virus-rep1b\_R1.fastq.gz | mut-virus-rep1b\_R2.fastq.gz |
| mut-virus-rep2b | SRR7694018 | mut-virus-rep2b\_R1.fastq.gz | mut-virus-rep2b\_R2.fastq.gz |
| mut-virus-rep3b | SRR7694003 | mut-virus-rep3b\_R1.fastq.gz | mut-virus-rep3b\_R2.fastq.gz |
| wt-virus-rep1b | SRR7693980 | wt-virus-rep1b\_R1.fastq.gz | wt-virus-rep1b\_R2.fastq.gz |
| wt-virus-rep3b | SRR7693984 | wt-virus-rep3b\_R1.fastq.gz | wt-virus-rep3b\_R2.fastq.gz |
| wt-virus-rep2b | SRR7693970 | wt-virus-rep2b\_R1.fastq.gz | wt-virus-rep2b\_R2.fastq.gz |
| mut-DNA-rep2 | SRR7694002 | mut-DNA-rep2\_R1.fastq.gz | mut-DNA-rep2\_R2.fastq.gz |
| mut-DNA-rep1 | SRR7693986 | mut-DNA-rep1\_R1.fastq.gz | mut-DNA-rep1\_R2.fastq.gz |
| mut-DNA-rep3 | SRR7694021 | mut-DNA-rep3\_R1.fastq.gz | mut-DNA-rep3\_R2.fastq.gz |
| wt-DNA-rep2 | SRR7693998 | wt-DNA-rep2\_R1.fastq.gz | wt-DNA-rep2\_R2.fastq.gz |
| wt-DNA-rep1 | SRR7693989 | wt-DNA-rep1\_R1.fastq.gz | wt-DNA-rep1\_R2.fastq.gz |
| wt-DNA-rep3 | SRR7694017 | wt-DNA-rep3\_R1.fastq.gz | wt-DNA-rep3\_R2.fastq.gz |
| mut-virus-rep1b-T20-8ug | SRR8097920 | mut-virus-rep1b-T20-8ug\_R1.fastq.gz | mut-virus-rep1b-T20-8ug\_R2.fastq.gz |
| mut-virus-rep2b-T20-8ug | SRR8097919 | mut-virus-rep2b-T20-8ug\_R1.fastq.gz | mut-virus-rep2b-T20-8ug\_R2.fastq.gz |
| mut-virus-rep3b-T20-8ug | SRR8097918 | mut-virus-rep3b-T20-8ug\_R1.fastq.gz | mut-virus-rep3b-T20-8ug\_R2.fastq.gz |

## Define samples and FASTQ\_files¶

In [3]:

```
R1fastqfilelist_df = pd.read_csv("./data/BG505_T20_samples.csv", header =0)
#display(HTML(R1fastqfilelist_df.to_html(index=False)))
```

## Process the FASTQ files to count the mutations for each sample¶

We used barcoded-subamplicon sequencing to obtain high accuracy during the Illumina deep sequencing. We therefore use the dms2\_batch\_bcsubamp program to analyze these data.

Running that program requires specifying a `--batchfile` that lists the samples, a wildtype `--refseq` to which we make alignments, and --alignspecs that tell us where the subamplicons should align.
The batch file that we specify is printed by the cell below. The alignment specs need to be exactly correct for the subamplicons to align. We also do some trimming of the reads using the `--R1trim` and `--R2trim` parameters.

The wildtype sequence of the BG505.W6.C2.T332N Env used in this experiment is in the file ./data/BG505.W6.C2.T332N\_env.fasta.
This sequence is based on GenBank accession number DQ208458.1, with the introduced T332N mutation to knock in the glycan commonly targeted by this class of bnAbs.

In [4]:

```
fastqdir = "./results/FASTQ_files/"
```

In [5]:

```
refseq = './data/BG505.W6.C2.T332N_env.fasta'

# define subamplicon alignment specifications
alignspecs = ' '.join(['87,375,39,36', 
                       '376,666,36,39',
                       '663,954,33,41',
                       '955,1228,33,37',
                       '1228,1527,34,35',
                       '1527,1815,32,39',
                       '1816,2098,36,41'])

# counts and alignments placed in this directory
countsdir = os.path.join(resultsdir, 'codoncounts')
if not os.path.isdir(countsdir):
    os.mkdir(countsdir)
    
# write sample information to a batch file for dms2_batch_bcsubamplicons
countsbatchfile = os.path.join(countsdir, 'batch.csv')
print("Here is the batch file that we write to CSV format to use as input:")
display(HTML(R1fastqfilelist_df[['name', 'R1']].to_html(index=False)))
R1fastqfilelist_df[['name', 'R1']].to_csv(countsbatchfile, index=False)

print('\nNow running dms2_batch_bcsubamp...')
log = !dms2_batch_bcsubamp \
        --batchfile {countsbatchfile} \
        --refseq {refseq} \
        --alignspecs {alignspecs} \
        --outdir {countsdir} \
        --summaryprefix summary \
        --R1trim 200 \
        --R2trim 170 \
        --minq 15 \
        --fastqdir {fastqdir} \
        --ncpus {ncpus} \
        --use_existing {use_existingalignment} 
print("Completed dms2_batch_bcsubamp.")
```

```
Here is the batch file that we write to CSV format to use as input:
```

| name | R1 |
| --- | --- |
| mut-virus-rep1b | mut-virus-rep1b\_R1.fastq.gz |
| mut-virus-rep2b | mut-virus-rep2b\_R1.fastq.gz |
| mut-virus-rep3b | mut-virus-rep3b\_R1.fastq.gz |
| wt-virus-rep1b | wt-virus-rep1b\_R1.fastq.gz |
| wt-virus-rep3b | wt-virus-rep3b\_R1.fastq.gz |
| wt-virus-rep2b | wt-virus-rep2b\_R1.fastq.gz |
| mut-DNA-rep2 | mut-DNA-rep2\_R1.fastq.gz |
| mut-DNA-rep1 | mut-DNA-rep1\_R1.fastq.gz |
| mut-DNA-rep3 | mut-DNA-rep3\_R1.fastq.gz |
| wt-DNA-rep2 | wt-DNA-rep2\_R1.fastq.gz |
| wt-DNA-rep1 | wt-DNA-rep1\_R1.fastq.gz |
| wt-DNA-rep3 | wt-DNA-rep3\_R1.fastq.gz |
| mut-virus-rep1b-T20-8ug | mut-virus-rep1b-T20-8ug\_R1.fastq.gz |
| mut-virus-rep2b-T20-8ug | mut-virus-rep2b-T20-8ug\_R1.fastq.gz |
| mut-virus-rep3b-T20-8ug | mut-virus-rep3b-T20-8ug\_R1.fastq.gz |

```
Now running dms2_batch_bcsubamp...
Completed dms2_batch_bcsubamp.
```

Now we look at the summary plots created by `dms2_batch_bcsubamp`.
All of these files are found in the directory specified by --outdir, and with the prefix specified by summaryprefix.
So we define them using this plot prefix plus the suffix for each plot.
Note that these files all refer to sites in sequential 1, 2, ... numbering of the BG505 sequence.

In [6]:

```
countsplotprefix = os.path.join(countsdir, 'summary')
```

The `*_readstats.pdf` plot below shows the statistics on the reads. This plot shows that most of the reads were retained, and a small fraction discarded because of low-quality barcodes. None failed the Illumina filter as those were already filtered out those when we downloaded from the SRA.

In [7]:

```
showPDF(countsplotprefix + '_readstats.pdf', width=600)
```

The `*_readsperbc.pdf` plot below shows how many times different barcodes were observed for each sample. Barcodes need to be observed multiple times to be useful for barcoded-subamplicon sequencing error correction.

In [8]:

```
showPDF(countsplotprefix + '_readsperbc.pdf')
```

The `*_bcstats.pdf` plot below shows statistics on the barcodes. Some of the barcodes had to be discarded because they had too few reads (these are the single-read barcodes in the plot above), a small fraction with adequate reads were not alignable, and the rest aligned to the Env gene properly.
This plot and the one above suggest that probably could have gotten additional depth by sequencing more, since then we would have had more barcodes with multiple reads.

In [9]:

```
showPDF(countsplotprefix + '_bcstats.pdf', width=600)
```

The `*_depth.pdf` plot below shows the depth (number of called codons) at each site in the gene.
For most of the samples, the depth across the gene is fairly uniform, indicating that the subamplicons were pooled fairly evenly.  
Note that some samples (in particular the mock samples) were intentionally sequenced to higher depth than the antibody-selected samples, as we expect to see more diversity in the mock samples.
Note that the gene was not sequenced past codon site 691, and so there is no coverage there.

In [10]:

```
showPDF(countsplotprefix + '_depth.pdf')
```

The `*_mutfreq.pdf` plot below shows the per-codon frequency of mutations at each site.
For each PGT151-selected sample, we see a few sites of clear peaks in mutation frequency.
These peaks tend to occur at the same sites in different replicates, and so presumably represent the sites where antibody-escape mutations are selected.
There are no such peaks for the mock sample since there is no antibody selection to favor specific mutations.
Note also that the gene was not mutagenized or sequenced past codon site 691, so there are no mutations there.

In [11]:

```
showPDF(countsplotprefix + '_mutfreq.pdf')
```

The `*_codonmuttypes.pdf` plot below shows the per-codon frequency of nonsynonymous, synonymous, and stop codon mutations across the entire gene. For the PGT151 selected samples, we see an overall increase in the per-codon mutation frequency due to very strong selection for variants with escape mutations.

In [12]:

```
showPDF(countsplotprefix + '_codonmuttypes.pdf', width=600)
```

The `*_codonntchanges.pdf` plot below shows same data as above but categorizes codon mutations by the number of nucleotides that are changed (e.g., ATG to AAG changes 1 nucleotide, ATG to AAC changes 2 nucleotides, and ATG to CAC changes 3 nucleotides).

In [13]:

```
showPDF(countsplotprefix + '_codonntchanges.pdf', width=600)
```

The `*_singlentchanges.pdf` plot below shows the frequency of each type of nucleotide change among only codon mutations with one nucleotide change. This plot is mostly useful to check if there is a large bias in which mutations appear. In particular, if you are getting oxidative damage (which causes G to T mutations) during the library preparation process, you will see a large excess of C to A or G to T mutations (or both). There is not much oxidative damage in the samples plotted below, which mostly have a fairly even distribution of nucleotide changes.

We do see that transitions (G <-> A and C <-> T) are a bit more common than most of the other types of mutations (all of which are transversions). This is expected, since PCR based sources of mutations are expected to preferentially introduce transitions.

This plot would also be important to examine any sign of APOBEC hypermutation (also G <-> A, with preferences for specific motifs) occuring in the HIV genome. One of the reasons we selected the SupT1.R5 cell line was because there is very little of the APOBEC proteins expressed in a subclone of SupT1 cells. In the viral samples, there does appear to be an increase in G to A and C to T mutations. However, we passaged WT virus in these cells and confirmed there were not signs of extensive APOBEC hypermutation in hotspot motifs via deep sequencing of env after passaging. Thus, we do not think there is extensive APOBEC hypermutation in our data, and this increase certain types of mutations in the viral samples is likely due to bias in the viral reverse transcription.

In [14]:

```
showPDF(countsplotprefix + '_singlentchanges.pdf', width=600)
```

## Renumber codon counts to HXB2 numbering¶

The standard numbering scheme for HIV Env is the HXB2 numbering scheme.
The file ./data/BG505\_to\_HXB2\_new.csv gives the mapping from sequential 1, 2, ... numbering of the BG505 protein sequence to the HXB2 numbering scheme.
This file was generated by aligning the HXB2 sequence taken from Genbank with BG505 and HXB2. I then manually edited this alignment such that an insertion is at 322a, not 317a, as described in Dingens et al 2018.

Insertions relative to HXB2 are given letter suffixes as described here.

Additionally, not all residues in Env were mutagenized.
The N-terminal signal peptide and the C-terminal cytoplasmic tail were excluded because they seem likely to affect expression level.
These sites are not listed in the renumbering file, and so are dropped when we do the re-numbering.

To do the re-numbering, we use the dms\_tools2.utils.renumberSites function from the dms\_tools Python API to create a new directory that contains all of the re-numbered files with the same name as in the original codon counts directory produced above.

In [15]:

```
renumberedcountsdir = os.path.join(resultsdir, 'renumberedcounts')
```

In [16]:

```
renumberfile = './data/BG505_to_HXB2_new.csv'

# renumbered counts will go here
renumberedcountsdir = os.path.join(resultsdir, 'renumberedcounts')

# counts files to renumber
countsfiles = glob.glob('{0}/*codoncounts.csv'.format(countsdir))

dms_tools2.utils.renumberSites(renumberfile, countsfiles, missing='drop', 
        outdir=renumberedcountsdir)
```

## Compute the differential selection and correlations between replicates¶

Now we compute the differential selection for each replicate, using the *wt-DNA* controls to estimate the error rates.
We also compute the mean and median differential selection across replicates.
Finally, we look at correlations between pairs of replicates in mutation and site differential selection, restricting to only positive differential selection.
We first create a batch file to use with dms2\_batch\_diffsel.
Note we make the **group** argument the antibody, the **name** argument the replicate, and assign the **sel**, **mock**, and **err** arguments based on the names used for the batch file when generating the counts files above with `dms2_batch_bcsubamp`.
By grouping replicates for the same antibody in the batch file, we tell dms2\_batch\_diffsel to analyze these together and take their mean and median.

In [17]:

```
#setup custom batch
codoncountslist = (glob.glob("./results/renumberedcounts/*_codoncounts.csv"))
codoncounts_df = pd.DataFrame(codoncountslist)
codoncounts_df.columns = ['codoncounts']
codoncounts_df["samplename"] = codoncounts_df['codoncounts']
codoncounts_df["samplename"].replace('_codoncounts.csv', '', regex=True, inplace=True)
codoncounts_df["samplename"].replace('./results/renumberedcounts/', '', regex=True, inplace=True)
codoncounts_df["samplename"].replace('_', '-', regex=True, inplace=True)
codoncounts_df["replicate"] = codoncounts_df["samplename"].str.split('-').str.get(2)
codoncounts_df["antibody"] = codoncounts_df["samplename"].str.split('-', expand=True, n=3).get(3)
#print(codoncounts_df)
sampledf = codoncounts_df.replace(to_replace='None', value="np.nan").dropna()
sampledf["group"] = sampledf['antibody']
sampledf["sel"] = sampledf["samplename"]
#sampledf["err"] = sampledf["replicate"]
#sampledf['err'] = 'wt-DNA-' + sampledf['err'].astype(str)
sampledf["mock"] = sampledf["replicate"]
sampledf['mock'] = 'mut-virus-' + sampledf['mock'].astype(str)

sampledf["name"] = sampledf['replicate'].astype(str) 
sampledf["mds_names"] = sampledf['group'].astype(str) + "-" + sampledf['name'].astype(str)


custombatch = sampledf[['group','name', "sel", "mock", "mds_names"]]
custombatch = custombatch.sort_values(by='group')
#print(custombatch)
```

In [18]:

```
# put diffsel values here
diffseldir = os.path.join(resultsdir, 'diffsel')
if not os.path.isdir(diffseldir):
    os.mkdir(diffseldir)
    

diffsel_wtDNActrl_dir = os.path.join(diffseldir, 'wtDNA_ctrl')
if not os.path.isdir(diffsel_wtDNActrl_dir):
    os.mkdir(diffsel_wtDNActrl_dir)
    

diffselbatch_wtDNActrl = custombatch.copy()
diffselbatch_wtDNActrl['err'] = 'wt-DNA-' + diffselbatch_wtDNActrl['name'].astype(str)
#diffselbatch_wtDNActrl['err'].replace('wt-virus', 'wt-DNA', regex=True, inplace=True)
diffselbatch_wtDNActrl['err'].replace('1b', '1', regex=True, inplace=True)
diffselbatch_wtDNActrl['err'].replace('2b', '2', regex=True, inplace=True)
diffselbatch_wtDNActrl['err'].replace('3b', '3', regex=True, inplace=True)


diffselbatchfile_wtDNActrl = os.path.join(diffsel_wtDNActrl_dir, 'batch.csv')
print("Here is the batch file that we write to CSV format to use as input:")
display(HTML(diffselbatch_wtDNActrl.to_html(index=False)))
diffselbatch_wtDNActrl.to_csv(diffselbatchfile_wtDNActrl, index=False)
```

```
Here is the batch file that we write to CSV format to use as input:
```

| group | name | sel | mock | mds\_names | err |
| --- | --- | --- | --- | --- | --- |
| T20-8ug | rep2b | mut-virus-rep2b-T20-8ug | mut-virus-rep2b | T20-8ug-rep2b | wt-DNA-rep2 |
| T20-8ug | rep3b | mut-virus-rep3b-T20-8ug | mut-virus-rep3b | T20-8ug-rep3b | wt-DNA-rep3 |
| T20-8ug | rep1b | mut-virus-rep1b-T20-8ug | mut-virus-rep1b | T20-8ug-rep1b | wt-DNA-rep1 |

In [19]:

```
log = !dms2_batch_diffsel \
        --summaryprefix summary \
        --batchfile {diffselbatchfile_wtDNActrl} \
        --outdir {diffsel_wtDNActrl_dir} \
        --indir {renumberedcountsdir} \
        --use_existing {use_existing}
```

In [20]:

```
import warnings
warnings.filterwarnings('ignore')
```

In [21]:

```
#custom functions:
#plot line plot! with overlay. 
#plot the line plot with overlays
import re
import os
import math
import natsort
#import pandas
import numpy
import scipy.stats
import matplotlib
matplotlib.use('pdf')
import matplotlib.pyplot as plt

from plotnine import *
# set ggplot theme
theme_set(theme_bw(base_size=12)) 

import seaborn


from dms_tools2 import CODONS, AAS, AAS_WITHSTOP
import dms_tools2.utils
COLOR_BLIND_PALETTE = ["#000000", "#E69F00", "#56B4E9", "#009E73",
                       "#F0E442", "#0072B2", "#D55E00", "#CC79A7"]

def plotSiteDiffSel_YLim(names, diffselfiles, plotfile, 
        diffseltype, highlighted_sites=None, maxcol=2, white_bg=False):
    """Plot site diffsel or fracsurvive along sequence.
    Despite the function name, this function can be used to
    plot either differential selection or fraction surviving.
    Args:
        `names` (list or series)
            Names of samples for which we plot statistics.
        `diffselfiles` (list or series)
            ``*sitediffsel.csv`` files from ``dms2_diffsel`` or
            ``*sitefracsurvive.csv`` files from ``dms2_fracsurvive``.
        `plotfile` (str)
            Name of created PDF plot file.
        `diffseltype` (str)
            Type of diffsel or fracsurvive to plot:
                - `positive`: positive sitediffsel
                - `total`: positive and negative sitediffsel
                - `max`: maximum mutdiffsel
                - `minmax`: minimum and maximum mutdiffsel
                - `avgfracsurvive`: total site fracsurvive
                - `maxfracsurvive`: max mutfracsurvive at site
        `maxcol` (int)
            Number of columns in faceted plot.
        `highlighted_sites` (list)
            Highlight sites of interest (passed in string format) in grey.
        `white_bg` (bool)
            Plots will have a white background with limited other formatting.
            
        DOCUMENT CHANGES
    """
    assert len(names) == len(diffselfiles) == len(set(names)) > 0
    assert os.path.splitext(plotfile)[1].lower() == '.pdf'

    diffsels = [pd.read_csv(f).assign(name=name) for (name, f) 
            in zip(names, diffselfiles)]
    assert all([set(diffsels[0]['site']) == set(df['site']) for df in 
            diffsels]), "diffselfiles not all for same sites"
    diffsel = pd.concat(diffsels, ignore_index=True)

    ylabel = 'differential selection'
    if diffseltype == 'positive':
        rename = {'positive_diffsel':'above'}
    elif diffseltype == 'total':
        rename = {'positive_diffsel':'above',
                  'negative_diffsel':'below'}
    elif diffseltype == 'max':
        rename = {'max_diffsel':'above'}
    elif diffseltype == 'minmax':
        rename = {'max_diffsel':'above',
                  'min_diffsel':'below'}
    elif diffseltype in ['avgfracsurvive', 'maxfracsurvive']:
        ylabel = 'fraction surviving'
        rename = {diffseltype:'above'}
    else:
        raise ValueError("invalid diffseltype {0}".format(diffseltype))
    diffsel = (diffsel.rename(columns=rename)
                      .melt(id_vars=['site', 'name'], 
                            value_vars=list(rename.values()),
                            value_name='diffsel',
                            var_name='direction')
                      )

    y_lim = diffsel['diffsel'].max() #get a maximum value used to plot the overlay
    # natural sort by site: https://stackoverflow.com/a/29582718
    diffsel = diffsel.reindex(index=natsort.order_by_index(
            diffsel.index, natsort.index_natsorted(diffsel.site,
            signed=True)))
    # now some manipulations to make site str while siteindex is int
    diffsel['site'] = diffsel['site'].apply(str)
    diffsel['siteindex'] = pd.Categorical(diffsel['site'],
            diffsel['site'].unique()).codes
    
    ncol = min(maxcol, len(names))
    nrow = math.ceil(len(names) / float(ncol))

    # make name a category to preserve order
    diffsel['name'] = diffsel['name'].astype('category', 
            categories=names)
    
    (xbreaks, xlabels) = dms_tools2.plot.breaksAndLabels(diffsel['siteindex'].unique(), 
            diffsel['site'].unique(), n=6)
    diffsel['highlight'] = diffsel['site'].isin(highlighted_sites) 
    diffsel['highlight'] =np.where(diffsel['highlight']==True , y_lim, 0)
    if white_bg:
        p = (ggplot(diffsel, aes(x='siteindex', y='diffsel',
                    color='direction', fill='direction'))
             + geom_bar(aes(y='highlight'), alpha=0.5, stat="identity", color="#d9d9d9", size=0.3, show_legend=False) 
             + geom_step(size=0.3)
             + xlab('site')
             + ylab(ylabel)
             + scale_x_continuous(breaks=xbreaks, labels=xlabels)
             + scale_color_manual(COLOR_BLIND_PALETTE)
             + scale_fill_manual(COLOR_BLIND_PALETTE)
             + guides(color=False)
             + theme(panel_background=element_rect(fill='white'),
                     axis_line_x=element_line(color='black'),
                     axis_line_y=element_line(color='black'),
                     panel_grid=element_blank(),
                     panel_border=element_blank(),
                     strip_background=element_blank()
                     )
            )
    else:
        p = (ggplot(diffsel, aes(x='siteindex', y='diffsel', color='direction'))
             + geom_bar(aes(y='highlight'), alpha=0.5, stat="identity", color="#d9d9d9", size=0.3, show_legend=False) 
             + geom_step(size=0.4)
             + xlab('site')
             + ylab(ylabel)
             + scale_x_continuous(breaks=xbreaks, labels=xlabels)
             + scale_color_manual(COLOR_BLIND_PALETTE)
             + guides(color=False)
             )
    if not ((len(names) == 1) and ((not names[0]) or names[0].isspace())):
        p += facet_wrap('~name', ncol=ncol)
    p += theme(figure_size=(4.6 * (0.3 + ncol), 1.9 * (0.2 + nrow)))
    p.save(plotfile, verbose=False)
    plt.close()
```

First, lets look at the `positive site differential` selection plotted across the length of the gene.

In [22]:

```
keysites = list(range(76,77)) + list(range(119, 122)) + list(range(207, 208)) + list(range(424, 437)) + list(range(544, 562))

# Env sites numbers are strings because some have trailing letters (e.g., 100A)
keysites = list(map(str, keysites))
```

In [23]:

```
#lineplots
names = ["T-20"]
diffselfiles = ["./results/diffsel/wtDNA_ctrl/summary_T20-8ug-mediansitediffsel.csv"]
plotfile = './results/diffsel/wtDNA_ctrl/T20_overlay_mediansitefracsurvive.pdf'
#we will be able to update the below command to using dms_tools2.plot.plotSiteDiffSel once it is part of dms_tools2 version used in BloomLab conda environement
plotSiteDiffSel_YLim(names, diffselfiles, plotfile, "positive", maxcol=1, highlighted_sites=keysites, white_bg=True)
showPDF(plotfile)

#now, a zoomed in view
```

Now, we will zoom in on the regions of interest highlighted in grey in the above plot.

In [24]:

```
import natsort
names = ["T20"]
diffselfiles = ["./results/diffsel/wtDNA_ctrl/summary_T20-8ug-medianmutdiffsel.csv"]

mutdiffsels = [] #list of csv files
for mutdiffsel, name in zip(diffselfiles, names): 
    pandadf = pd.read_csv(mutdiffsel)

    # read data frame from tidy CSV format to wide form
    df = dms_tools2.diffsel.tidyToWide(pandadf, valuecol='mutdiffsel')
    
    # only show positive values of mutdiffsel
    df[dms_tools2.AAS] = df[dms_tools2.AAS].clip(lower=0)
    
    #sort sites by 
    df = df.reindex(index=natsort.order_by_index(df.index,natsort.index_natsorted(df.site)))
        
    # add a column indicating what we should show
    df['show'] = df['site'].isin(keysites)
    
    
    # want to indicate wildtype along with site number, e.g, A512
    df['site'] = df['wildtype'] + df['site']

    # name of file to plot
    zoomlogoplot = os.path.join(diffsel_wtDNActrl_dir, '{0}_zoomed_diffsel.pdf'.format(name))
    #print(df)
    # plot and show
    dms_tools2.rplot.siteSubsetGGSeqLogo(
            logodata=df,
            chars=dms_tools2.AAS,
            plotfile=zoomlogoplot,
            width=8,
            height=2.5,
            yname='differential selection',
            ylimits=(0,40),
            )
    showPDF(zoomlogoplot)
```

Now, lets look at the correlations between biological replicates, and then the above line and logo plots for each individual replicate.

In [25]:

```
#correlations
print("Here is the correlation for the positive site differential selection:")
showPDF("./results/diffsel/wtDNA_ctrl/summary_T20-8ug-positivesitediffselcorr.pdf", width=500)
print("Here is the correlation for the mutation differential selection:")
showPDF("./results/diffsel/wtDNA_ctrl/summary_T20-8ug-mutdiffselcorr.pdf", width=500)
```

```
Here is the correlation for the positive site differential selection:
```

```
Here is the correlation for the mutation differential selection:
```

In [26]:

```
#linepolot across replicates 
names = ["T20_rep1_8ug", "T20_rep2_8ug", "T20_rep3_8ug"]
diffselfiles = ["./results/diffsel/wtDNA_ctrl/T20-8ug-rep1b_sitediffsel.csv", 
                "./results/diffsel/wtDNA_ctrl/T20-8ug-rep2b_sitediffsel.csv", 
                "./results/diffsel/wtDNA_ctrl/T20-8ug-rep3b_sitediffsel.csv"
               ]
plotfile = './results/diffsel/wtDNA_ctrl/T20_overlay_replicates_sitefracsurvive.pdf'

plotSiteDiffSel_YLim(names, diffselfiles, plotfile, "positive", maxcol=1, highlighted_sites=keysites, white_bg=True)
showPDF(plotfile)
```

In [27]:

```
#and now we plot the zoomed in view across replicates 
names = ["T20_rep1_8ug", "T20_rep2_8ug", "T20_rep3_8ug"]
diffselfiles = ["./results/diffsel/wtDNA_ctrl/T20-8ug-rep1b_mutdiffsel.csv", 
                "./results/diffsel/wtDNA_ctrl/T20-8ug-rep2b_mutdiffsel.csv", 
                "./results/diffsel/wtDNA_ctrl/T20-8ug-rep3b_mutdiffsel.csv"
               ]
for mutdiffsel, name in zip(diffselfiles, names): 
    pandadf = pd.read_csv(mutdiffsel)
    # read data frame from tidy CSV format to wide form
    df = dms_tools2.diffsel.tidyToWide(pandadf, valuecol='mutdiffsel')
    # only show positive values of mutdiffsel
    df[dms_tools2.AAS] = df[dms_tools2.AAS].clip(lower=0)
    #sort sites by 
    df = df.reindex(index=natsort.order_by_index(df.index,natsort.index_natsorted(df.site)))
    # add a column indicating what we should show
    df['show'] = df['site'].isin(keysites)
    # want to indicate wildtype along with site number, e.g, A512
    df['site'] = df['wildtype'] + df['site']
    # name of file to plot
    zoomlogoplot = os.path.join(diffsel_wtDNActrl_dir, '{0}_zoomed_diffsel.pdf'.format(name))
    # plot and show
    dms_tools2.rplot.siteSubsetGGSeqLogo(
            logodata=df,
            chars=dms_tools2.AAS,
            plotfile=zoomlogoplot,
            width=8,
            height=2.5,
            yname='differential selection',
            ylimits=(0,55),
            )
    showPDF(zoomlogoplot)
```

## Make logo plots visualizing the differential selection¶

We now plot the entirety of the resistance profile across Env using dms2\_logoplot.

In [28]:

```
groups = diffselbatch_wtDNActrl['group'].unique()
```

In [29]:

```
for antibody in groups:

    mutdiffsel = os.path.join(diffsel_wtDNActrl_dir, 'summary_{0}-medianmutdiffsel.csv'.format(antibody))
    # now create the logo plot with the overlay
    #scale bar unit is maximum effect
    mutdiffseldf = pd.read_csv(mutdiffsel)
    scaleunit = '10'
    #scaleunit = '{0:.1g}'.format(mutdiffseldf['mutdiffsel'].max())
    scalelabel = '"differential selection = {0}"'.format(scaleunit)
    logoplot = os.path.join(diffsel_wtDNActrl_dir, '{0}-median_diffsel.pdf'.format(antibody))
    logoname = '{0}-median'.format(antibody)
    print(logoplot)
    print("\nCreating logo plot for {0} from {1}".format(antibody, mutdiffsel))
    log = !dms2_logoplot \
            --diffsel {mutdiffsel} \
            --name {logoname} \
            --outdir {diffsel_wtDNActrl_dir} \
            --restrictdiffsel positive \
            --sepline no \
            --nperline 84 \
            --overlay1 {mutdiffsel} wildtype wildtype \
            --scalebar {scaleunit} {scalelabel} \
            --underlay yes \
            --use_existing no
    showPDF(logoplot)
    
for antibody in groups:

    mutdiffsel = os.path.join(diffsel_wtDNActrl_dir, 'summary_{0}-meanmutdiffsel.csv'.format(antibody))
    # now create the logo plot with the overlay
    #scale bar unit is maximum effect
    mutdiffseldf = pd.read_csv(mutdiffsel)
    scaleunit = '10'
    #scaleunit = '{0:.1g}'.format(mutdiffseldf['mutdiffsel'].max())
    scalelabel = '"differential selection = {0}"'.format(scaleunit)
    logoplot = os.path.join(diffsel_wtDNActrl_dir, '{0}-mean_diffsel.pdf'.format(antibody))
    logoname = '{0}-mean'.format(antibody)
    print(logoplot)
    print("\nCreating logo plot for {0} from {1}".format(antibody, mutdiffsel))
    log = !dms2_logoplot \
            --diffsel {mutdiffsel} \
            --name {logoname} \
            --outdir {diffsel_wtDNActrl_dir} \
            --restrictdiffsel positive \
            --sepline no \
            --nperline 84 \
            --overlay1 {mutdiffsel} wildtype wildtype \
            --scalebar {scaleunit} {scalelabel} \
            --underlay yes \
            --use_existing {use_existing}
    #showPDF(logoplot)
```

```
./results/diffsel/wtDNA_ctrl/T20-8ug-median_diffsel.pdf

Creating logo plot for T20-8ug from ./results/diffsel/wtDNA_ctrl/summary_T20-8ug-medianmutdiffsel.csv
```

```
./results/diffsel/wtDNA_ctrl/T20-8ug-mean_diffsel.pdf

Creating logo plot for T20-8ug from ./results/diffsel/wtDNA_ctrl/summary_T20-8ug-meanmutdiffsel.csv
```

## Compute the fraction surviving¶

Now we compute the fraction surviving, using the *wt-virus* controls to estimate the error rates. This caluclation takes into account the level of drug selection, found in the input file below.

**These data are not included in the paper, but we include it in this analysis in case there is interest in also anlyzing these data. Overall, these data appear slightly cleaner, but emphasize the low-level escape outside of the enfuvirtide binding site less. That is partially because the `differential selection` is log-transformed, while the `fraction surviving` is not (thus, differential selection places more weight/emphasis on smaller values).** We used the differential selection to emphasize this allosteric resistance more. Of note, we validated that this resistance indeed affects enfuvirtide sensitivity, and other have previously shown that *some* mutations to this region can affect enfuviritide sensitivity Reeves et al 2002, Herschhorn et al 2017.

This csv file contains the fraction remaining infectious for each enfuvirtide selected sample, as quatified using pol qPCR and computed based on a standard curve of infecting cells with dilutions of mutant virus (library and experiment specific), with dilutiuons ranging from 0.1 to .0001.

We first create a batch file to use with dms2\_batch\_fracsurvive.
Note we make the **group** argument the antibody, the **name** argument the replicate, and assign the **sel**, **mock**, and **err** arguments based on the names used for the batch file when generating the counts files above with `dms2_batch_bcsubamp`.
By grouping replicates for the same antibody in the batch file, we tell dms2\_batch\_fracsurvive to analyze these together and take their mean and median.

Here, we are using sequencing of **wt-DNA** as an error control.

In [30]:

```
fracsurvivedir = os.path.join(resultsdir, 'fracsurvive')
if not os.path.isdir(fracsurvivedir):
    os.mkdir(fracsurvivedir)
    
fracsurviveaboveavgdir = os.path.join(resultsdir, 'fracsurviveaboveavg')
if not os.path.isdir(fracsurviveaboveavgdir):
    os.mkdir(fracsurviveaboveavgdir)
```

In [31]:

```
#set up fracsurvivebatch
qPCRdata = pd.read_csv('./data/BG505_qPCR_T20.csv')
qPCRdata["sel"].replace('BG505-', '', regex=True, inplace=True)

codoncountslist = (glob.glob("./results/renumberedcounts/*_codoncounts.csv"))
codoncounts_df = pd.DataFrame(codoncountslist)
codoncounts_df.columns = ['codoncounts']
codoncounts_df["samplename"] = codoncounts_df['codoncounts']
codoncounts_df["samplename"].replace('_codoncounts.csv', '', regex=True, inplace=True)
codoncounts_df["samplename"].replace('./results/renumberedcounts/', '', regex=True, inplace=True)
codoncounts_df["samplename"].replace('_', '-', regex=True, inplace=True)
codoncounts_df["replicate"] = codoncounts_df["samplename"].str.split('-').str.get(2)
codoncounts_df["antibody"] = codoncounts_df["samplename"].str.split('-', expand=True, n=3).get(3)
codoncounts_df
sampledf = codoncounts_df.replace(to_replace='None', value="np.nan").dropna()
sampledf["group"] = sampledf['antibody']
sampledf["sel"] = sampledf["samplename"]
sampledf["err"] = sampledf["replicate"]
sampledf['err'] = 'wt-DNA-' + sampledf['err'].astype(str)
sampledf['err'].replace('1b', '1', regex=True, inplace=True)
sampledf['err'].replace('2b', '2', regex=True, inplace=True)
sampledf['err'].replace('3b', '3', regex=True, inplace=True)

sampledf["mock"] = sampledf["replicate"]
sampledf['mock'] = 'mut-virus-' + sampledf['mock'].astype(str)

sampledf["name"] = sampledf['replicate'].astype(str) 
sampledf["mds_names"] = sampledf['group'].astype(str) + "-" + sampledf['name'].astype(str)


fracsurvivebatch = sampledf[['group','name', "sel", "mock", "err", "mds_names"]]
fracsurvivebatch = fracsurvivebatch.sort_values(by='group')

fracsurvivebatch = pd.merge(fracsurvivebatch, qPCRdata[['sel', 'libfracsurvive']], 
        left_on = 'sel', right_on= 'sel') #, suffixes=('', '_y')).drop('name_y', axis=1)

fracsurvivebatchfile = os.path.join(fracsurvivedir, 'batch.csv')
print("Here is the batch input that we write to the CSV file {0}:".format(fracsurvivebatchfile))
display(HTML(fracsurvivebatch.to_html(index=False)))
fracsurvivebatch.to_csv(fracsurvivebatchfile, index=False, encoding='utf-8')
```

```
Here is the batch input that we write to the CSV file ./results/fracsurvive/batch.csv:
```

| group | name | sel | mock | err | mds\_names | libfracsurvive |
| --- | --- | --- | --- | --- | --- | --- |
| T20-8ug | rep2b | mut-virus-rep2b-T20-8ug | mut-virus-rep2b | wt-DNA-rep2 | T20-8ug-rep2b | 0.001510 |
| T20-8ug | rep3b | mut-virus-rep3b-T20-8ug | mut-virus-rep3b | wt-DNA-rep3 | T20-8ug-rep3b | 0.004357 |
| T20-8ug | rep1b | mut-virus-rep1b-T20-8ug | mut-virus-rep1b | wt-DNA-rep1 | T20-8ug-rep1b | 0.007778 |

We now run `dms2_batch_survive` twice with the following difference:

```
1. First we run it simply computing the fraction surviving for each mutation.
2. Then we run it with the --aboveavg yes option to compute the fraction surviving for each mutation above the overall library average.
```

Note how the results for these two different runs are output to two different subdirectories.

In [32]:

```
for (arg_aboveavg, outdir) in [('', fracsurvivedir), ('--aboveavg yes', fracsurviveaboveavgdir)]:
    print("\nRunning dms2_batch_fracsurvive {0}and writing output to {1}".format(
            {'':'', '--aboveavg yes':'with `--aboveavg yes` '}[arg_aboveavg], outdir))
    log = !dms2_batch_fracsurvive \
            --summaryprefix summary \
            --batchfile {fracsurvivebatchfile} \
            --outdir {outdir} \
            --indir {renumberedcountsdir} \
            --use_existing {use_existing} \
            {arg_aboveavg} 
    print("Completed run.")
```

```
Running dms2_batch_fracsurvive and writing output to ./results/fracsurvive
Completed run.

Running dms2_batch_fracsurvive with `--aboveavg yes` and writing output to ./results/fracsurviveaboveavg
Completed run.
```

Now, we quickly plot the data to visualise the `fraction surviving` data.

In [33]:

```
groups = fracsurvivebatch['group'].unique()
for antibody in groups:
    
    mutdiffsel = os.path.join(fracsurviveaboveavgdir, 'summary_{0}-medianmutfracsurvive.csv'.format(antibody))
    logoplot = os.path.join(fracsurviveaboveavgdir, '{0}-median_fracsurvive.pdf'.format(antibody))
        #scale bar unit is maximum effect
    mutdiffseldf = pd.read_csv(mutdiffsel)
    scaleunit = '{0:.1g}'.format(mutdiffseldf['mutfracsurvive'].max())
    scalelabel = '"fraction surviving above avg = {0}"'.format(scaleunit)
    logoname = '{0}-median'.format(antibody)
    print("Creating logo plot for {0} from {1}".format(antibody, mutdiffsel))
    log = !dms2_logoplot \
            --fracsurvive {mutdiffsel} \
            --name {logoname} \
            --outdir {fracsurviveaboveavgdir} \
            --sepline no \
            --nperline 84 \
            --overlay1 {mutdiffsel} wildtype wildtype \
            --scalebar {scaleunit} {scalelabel} \
            --underlay yes \
            --use_existing {use_existing}
    #showPDF(logoplot)
    
    
    
    mutdiffsel = os.path.join(fracsurvivedir, 'summary_{0}-medianmutfracsurvive.csv'.format(antibody))
    logoplot = os.path.join(fracsurvivedir, '{0}-median_fracsurvive.pdf'.format(antibody))
        #scale bar unit is maximum effect
    mutdiffseldf = pd.read_csv(mutdiffsel)
    scaleunit = '{0:.1g}'.format(mutdiffseldf['mutfracsurvive'].max())
    scalelabel = '"fraction surviving = {0}"'.format(scaleunit)
    logoname = '{0}-median'.format(antibody)
    print("Creating logo plot for {0} from {1}".format(antibody, mutdiffsel))
    log = !dms2_logoplot \
            --fracsurvive {mutdiffsel} \
            --name {logoname} \
            --outdir {fracsurvivedir} \
            --sepline no \
            --nperline 84 \
            --overlay1 {mutdiffsel} wildtype wildtype \
            --scalebar {scaleunit} {scalelabel} \
            --underlay yes \
            --use_existing {use_existing}
    #showPDF(logoplot)
```

```
Creating logo plot for T20-8ug from ./results/fracsurviveaboveavg/summary_T20-8ug-medianmutfracsurvive.csv
Creating logo plot for T20-8ug from ./results/fracsurvive/summary_T20-8ug-medianmutfracsurvive.csv
```

In [34]:

```
showPDF("./results/fracsurviveaboveavg/T20-8ug-median_fracsurvive.pdf")
```

In [35]:

```
names = ["T-20"]
diffselfiles = ["./results/fracsurviveaboveavg/summary_T20-8ug-mediansitefracsurvive.csv", 
               ]

plotfile = './results/fracsurviveaboveavg/T20_Fracsurviveaboveavg_Overlay.pdf'

plotSiteDiffSel_YLim(names, diffselfiles, plotfile, "avgfracsurvive", maxcol=1, highlighted_sites=keysites,  white_bg=True)
showPDF(plotfile)
```

In [36]:

```
import natsort
#diffsel_wtDNActrl_dir
names = ["T20"]
diffselfiles = ["./results/fracsurviveaboveavg/summary_T20-8ug-medianmutfracsurvive.csv"]


mutdiffsels = [] #list of csv files
for mutdiffsel, name in zip(diffselfiles, names): 
    pandadf = pd.read_csv(mutdiffsel)

    # read data frame from tidy CSV format to wide form
    df = dms_tools2.diffsel.tidyToWide(pandadf, valuecol='mutfracsurvive')
    
    # only show positive values of mutdiffsel
    df[dms_tools2.AAS] = df[dms_tools2.AAS].clip(lower=0)
    
    #sort sites by 
    df = df.reindex(index=natsort.order_by_index(df.index,natsort.index_natsorted(df.site)))
    
    # add a column indicating what we should show
    df['show'] = df['site'].isin(keysites)
    
    
    # want to indicate wildtype along with site number, e.g, A512
    df['site'] = df['wildtype'] + df['site']
    # name of file to plot
    zoomlogoplot = os.path.join(fracsurviveaboveavgdir, '{0}_zoomed_fracsurviveaboveavg.pdf'.format(name))

    # plot and show
    dms_tools2.rplot.siteSubsetGGSeqLogo(
            logodata=df,
            chars=dms_tools2.AAS,
            plotfile=zoomlogoplot,
            width=8,
            height=2.5,
            yname='frac survive above avg',
            ylimits=(0,.5),
            )
    showPDF(zoomlogoplot)
```
